# Atherosclerosis Progression Evaluation in Coronary Arteries by Computed Tomography Compared to Peripheral Vessels Using 3D Ultrasonography

**DOI:** 10.3390/jcm15093546

**Published:** 2026-05-06

**Authors:** Maria Noflatscher, Michael Schreinlechner, Philip Sommer, Julian Freiberger, Fabian Plank, Pietro G. Lacaita, Markus Theurl, Rudolf Kirchmair, Axel Bauer, Gudrun Feuchtner, Peter Marschang

**Affiliations:** 1 Department of Internal Medicine III (Cardiology, Angiology), Medical University of Innsbruck, Anichstr. 35, 6020 Innsbruck, Austriajulian.freiberger@student.i-med.ac.at (J.F.); peter.marschang@sabes.it (P.M.); 2 Department of Radiology, Medical University of Innsbruck, Anichstr. 35, 6020 Innsbruck, Austria; 3 Department of Internal Medicine, Central Hospital of Bolzano (SABES-ASDAA), Via Lorenz Boehler 5, 39100 Bolzano, Italy

**Keywords:** atherosclerosis, coronary artery calcium score, ultrasonography, Doppler, computed tomography, plaque, atherosclerotic, disease progression, risk assessment

## Abstract

**Background:** Cardiovascular disease remains the leading cause of mortality worldwide. In recent years, several non-invasive imaging techniques have been introduced to improve the assessment of atherosclerotic burden and cardiovascular risk, including computed tomography and vascular ultrasound. Three-dimensional ultrasound has emerged as a promising method for the non-invasive quantification of plaque volume in peripheral arteries. **Methods:** In this prospective single-centre study, 63 patients with low to moderate cardiovascular risk (6–20% according to the Framingham Risk Score) were included. All participants underwent baseline examinations, and 38 patients completed follow-up after a mean period of 2 years. The assessment included coronary artery calcium scoring (CACS) by computed tomography and three-dimensional ultrasound to measure peripheral plaque volume in carotid and femoral arteries. Baseline values and progression of coronary and peripheral atherosclerosis were analysed using Spearman’s correlation. **Results:** Sixty-three patients were included in the final analysis. A significant correlation was observed between baseline CACS and peripheral plaque volume (r = 0.32; *p* = 0.010). In addition, the progression of coronary calcium scores was significantly associated with the progression of peripheral plaque volume (r = 0.34; *p* = 0.037). **Conclusions:** Coronary artery calcium is significantly associated with peripheral plaque burden, both at baseline and over time. The parallel progression of coronary and peripheral atherosclerosis suggests that peripheral plaque volume assessed by 3D ultrasound may serve as a surrogate marker for coronary disease progression.

## 1. Introduction

Atherosclerosis is a complex and systemic vascular condition that affects multiple arterial territories, including both coronary and peripheral vessels. It remains one of the principal contributors to mortality in industrialised regions. In clinical practice, cardiovascular risk estimation is commonly based on established scoring systems such as the ESC-SCORE and the Framingham Risk Score [1,2,3].

In recent decades, several non-invasive approaches have been developed to assess atherosclerotic burden and improve individual risk evaluation. Among these, computed tomography and ultrasound play a central role. Ultrasound is particularly advantageous due to its wide availability, low cost, and absence of radiation exposure. Numerous studies have investigated ultrasound-based markers such as intima–media thickness and plaque characteristics, especially in carotid arteries [4].

Established parameters in the evaluation of atherosclerotic wall changes include the intima–media thickness (IMT), different plaque scores and the plaque echogenicity [5,6,7,8]. However, a meta-analysis showed that IMT as a predictive parameter has few to no advantages in assessing individual cardiovascular risk compared to risk calculation based on the FRS [9].

The deposition of calcium in the coronary arteries, which can be measured as the coronary artery calcium score (CAC), is an important parameter for the estimation of atherosclerotic changes [10]. Baseline measurements of CAC can predict future cardiac events with a low radiation exposure (about 1 mSv) [10].

In particular, CAC determinations show a very high negative predictive value. With an Agatston score of 0 a significant coronary artery stenosis is ruled out with a very high degree of certainty [11]. This prognostic benefit led to a IIb recommendation in both the American and European Society of Cardiology guidelines for CAC testing in the risk evaluation of patients with intermediate cardiovascular risk [10,12]. According to the ESC guidelines, CAC testing can be considered in patients with low to intermediate risk.

The use of three-dimensional (3D) ultrasound for volumetric determination of plaque burden may be superior in diagnostic value to the evaluation of IMT alone. Baber et al. evaluated the predictive value of 3D plaque volumetry in the carotid arteries in a study [13]. The plaque burden in the carotid arteries and the CAC were initially determined in patients using 3D volumetry. The number of cardiovascular events in this patient group was observed over a median follow-up period of 2.7 years. 3D plaque volumetry has been shown to increase the predictive value over traditional risk factors and FRS and is comparable to CAC calculated by CT.

Also, current ESC guidelines underline the clinical relevance of carotid ultrasound for the detection of subclinical atherosclerosis and its contribution to improved cardiovascular risk stratification, facilitating the identification of patients at risk for coronary artery disease [14].

Despite the growing use of non-invasive imaging techniques for the assessment of atherosclerosis, important gaps remain in understanding the relationship between coronary and peripheral vascular disease. While coronary artery calcium is an established marker of coronary atherosclerosis and peripheral plaque burden can be assessed by ultrasound, there is limited evidence directly comparing these two modalities within the same population. In particular, data on the longitudinal progression of atherosclerosis across different vascular territories are scarce.

Therefore, the present study aimed to investigate the relationship between coronary artery calcification and peripheral plaque volume, as well as their progression over time, to determine whether peripheral plaque assessment may serve as a surrogate marker for coronary atherosclerosis.

## 2. Methods

### 2.1. Study Population

Participants were recruited from a prospective, single-centre observational cohort study investigating the relationship between atherosclerotic plaque volume, intima–media thickness, and soluble P-selectin (ClinicalTrials.gov identifier: NCT01895725), as previously reported [15,16,17]. These individuals were invited to take part in a dedicated sub-study (Atherosclerosis-Progression in coronary arteries compared to peripheral arteries, APRICOTS; ClinicalTrials.gov identifier: NCT03164174). Within this sub-study, additional coronary CT imaging was performed to evaluate and compare the extent and temporal progression of atherosclerotic changes in peripheral arteries with those occurring in the coronary circulation.

The original cohort comprised 443 patients aged between 30 and 85 years who presented with at least one cardiovascular risk factor—such as arterial hypertension, smoking, hyperlipidaemia, diabetes mellitus, or a positive family history of cardiovascular disease (CVD)—or with established vascular disease, including coronary artery disease (CAD), cerebrovascular disease (CBVD), or peripheral arterial disease (PAD). Detailed baseline characteristics as well as the inclusion and exclusion criteria of the primary study have been described previously [15]. For the APRICOTS sub-study, additional exclusion criteria were applied, including prior myocardial revascularisation procedures (e.g., coronary artery bypass grafting or percutaneous coronary intervention with stent implantation), a low (<6%) or high (>20%) Framingham Risk Score, pregnancy or breastfeeding, hyperthyroidism, and moderate to severe renal dysfunction (glomerular filtration rate <45 mL/min).

At baseline, all participants underwent cardiac CT imaging and three-dimensional ultrasound for the assessment of plaque volume. These examinations were repeated after a follow-up period of approximately two years. In parallel, EDTA blood samples were collected from peripheral venous blood alongside routine laboratory analyses. At follow-up, participants additionally underwent repeat coronary CT imaging, ultrasound examination, ankle–brachial index measurement, and an interim medical history focused on cardiovascular events.

The study protocol was approved by the ethics committee of the Medical University of Innsbruck (protocol numbers UN5048 and AN2016-0188) and was conducted in accordance with the Declaration of Helsinki. Written informed consent was obtained from all participants prior to enrolment.

### 2.2. Coronary CT Examination

During the CT examination, CAC was examined with standardised parameters using a 128-slice dual-source computed tomography (CT) system (Somatom Flash or Drive, Siemens Healthineers, Erlangen, Germany: Detector collimation 2 × 64 × 0.6 mm, 0.28 s rotation time, 120 kV, 3 mm image thickness (1.5 increment), prospective ECG gating triggered into the diastolic phase) and the CAC was calculated (Agaston score) using a post-processing software (Syngovia, Siemens Healthineers, Erlangen, Germany) for all coronary vessels (LCA; RCA).

Subsequently, a contrast-enhanced CT angiography (128-slice dual-source CT Flash, Siemens, 128 × 0.6 mm, 0.28 s gantry rotation time) of the coronary vessels with iodine-containing contrast medium (60–100 mL Ultravist 370) was performed with the aid of ECG gating (prospective, high-pitch or sequential) to exclude significant coronary stenoses. Retrospective gating was used in patients with absolute arrhythmia.

All CT images were analysed by experienced investigators who were blinded to the clinical and ultrasound data. Coronary artery calcium scoring was performed according to the Agatston method, applying a standard threshold of 130 Hounsfield units. Quality control procedures were implemented to ensure consistency across measurements.

### 2.3. Ultrasound Imaging

Peripheral plaques were defined in accordance with the Mannheim consensus criteria [18], including focal arterial wall thickening extending 0.5 mm into the lumen or exceeding predefined thresholds relative to IMT (>50% of surrounding IMT) or total lesion (>1.5 mm from the media–adventitia interface to the intima–lumen interface).

Ultrasound examinations were conducted using a standardised protocol with a Philips iU22 system (Philips, Amsterdam, The Netherlands) [15]. Three-dimensional imaging was applied to quantify plaque volume in carotid and femoral arteries, including the carotid bifurcation and adjacent arterial segments. Plaque volume values from both sides were combined to calculate total peripheral plaque burden.

All ultrasound examinations were performed by trained operators with experience in vascular imaging. To ensure reproducibility, measurements were conducted according to a standardised protocol, and intra-observer variability was minimised by repeated assessments in selected cases.

### 2.4. Statistical Analysis

All statistical analyses were performed using SPSS software (version 27.0.1, IBM, Armonk, NY, USA) [19]. Data distribution was assessed using the Kolmogorov–Smirnov test. Continuous variables are presented as mean ± standard deviation for normally distributed data or as median with interquartile range otherwise.

Group comparisons were performed using the Mann–Whitney U test for continuous variables and the chi-square test for categorical data. Bootstrapping methods were applied to estimate confidence intervals. The progression of CAC values and plaque volume over time were analysed using Spearman’s correlation. The correlation coefficients of the progression of coronary and peripheral atherosclerosis were then compared using Fisher’s Z transformation.

Statistical significance was defined as a *p*-value below 0.05.

For sample size calculation, a type I error of 5% and a Spearman correlation coefficient of r = 0.4, based on progression data from previous studies [20,21,22], were assumed. To achieve a statistical power of 80% and considering an anticipated dropout rate of 10%, a total of 55 patients needed to be included.

## 3. Results

**Figure 1:**
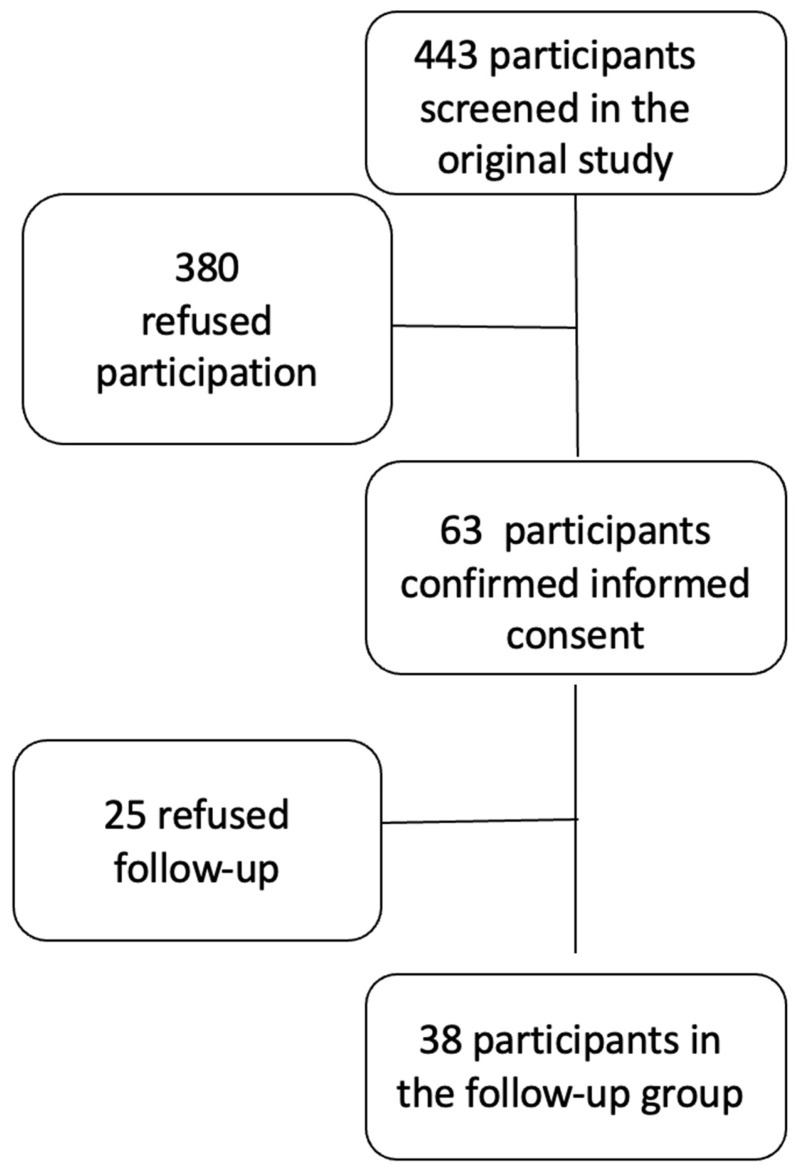
Flow chart of study participants.

A total of 63 subjects of the original study cohort agreed to participate in the APRICOT study shown in the flow chart in Figure 1.

The study population was divided into a baseline group (*n* = 25) and a follow-up group (*n* = 38) according to the number of CT examinations. Subjects in the baseline group underwent only the baseline CT examination, while participants in the follow-up group underwent both the baseline examination and a follow-up CT examination after an average of 24.1 months. The characteristics of the study population and the two groups are shown in Table 1. The mean age of all subjects at inclusion was 61.9 years [IQR 55.34–68.72]. In the baseline group, the median age was 60.2 years [IQR 55.67–71.22], while in the follow-up group it was 63.8 years [IQR 54.61–68.33]. In addition, 41.3% (*n* = 26) of the study population are female. In the baseline group, 32% (*n* = 8) are female, and in the follow-up group 47.4% (*n* = 18) are female. No statistically significant differences were found between the two groups at baseline. Furthermore, 12.7% of the patients in the baseline and follow-up groups achieved target (70 mg/dL) LDL cholesterol values.

The median CAC in the entire study population at the time of baseline examination was 73.3 Agatston units [IQR 2.80–324.40]. In the baseline group a CAC score of 55.4 Agatston units [IQR 3.70–317.45] could be determined, whereas the follow-up group had a CAC score of 74.7 Agatston units [IQR 0.90–340.95] at baseline. No statistically significant difference in the measured parameters was detected between the two groups at the time of baseline examination, Table 2.

The median peripheral plaque volume of all patients was 270.0 mm^3^ [IQR 99.00–477.00]. The peripheral plaque volume in the baseline group was 280 mm^3^, whereas in the first examination of the follow-up group it was 269.5 mm^3^ [IQR 89.75–479.00]. No statistically significant difference was found in the peripheral plaque volume between the two groups.

**Figure 2:**
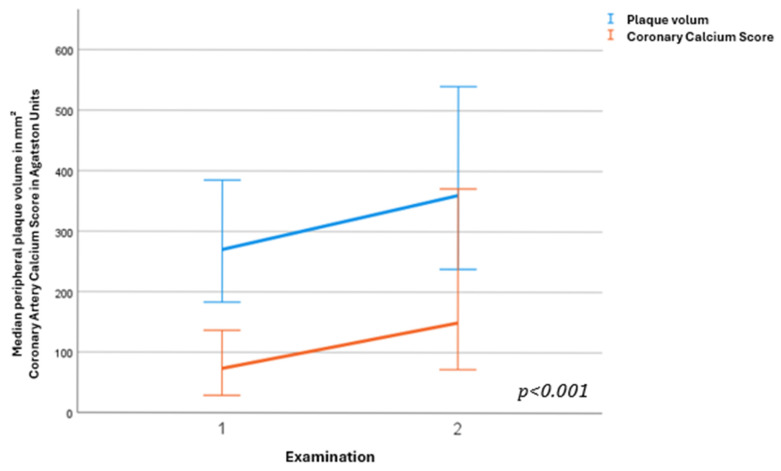
Progression of coronary artery calcium score and peripheral plaque volume. Over 2 years, mean plaque volume (in blue) increased by a median of 81.5 mm^3^, while coronary calcium (in orange) increased by a median of 68.5 Agatston units.

To compare the changes in the two variables CAC and combined plaque volume from baseline to follow-up, a progression analysis was performed. As described previously [17] progression was defined as the differences in the absolute values of the two measures at the two study time points. Figure 2 summarises the statistical difference between the two examinations and the calculated progressions. The CAC increased by a median of 68.5 Agatston units [IQR 8.35–152.33] between the two examinations, while the combined plaque volume increased by a median of 81.5 mm [IQR 36.25–131.00]. Thus, the follow-up values were statistically significantly different from the baseline values (*p* < 0.001).

Furthermore, a significant correlation (r = 0.32; *p* = 0.010) was found between the baseline CAC levels and the peripheral plaque volume of carotid and femoral arteries. Similarly, a significant correlation (r = 0.34; *p* = 0.037) was found between the progression levels of the peripheral plaque volume and the CAC values.

Overall, both baseline and longitudinal analyses consistently demonstrated a significant association between coronary artery calcium and peripheral plaque volume. These findings were robust across the analysed subgroups and remained consistent when considering both absolute values and progression measures.

The results remained unchanged when patients with and without statin therapy were analysed separately.

## 4. Discussion

In the present study, we observed a significant association between peripheral plaque burden assessed by 3D ultrasound and coronary artery calcium measured by computed tomography, both at baseline and during follow-up. To our knowledge, the progression of coronary and peripheral arterial atherosclerosis has not been compared before using these imaging modalities.

Due to the current lack of studies, there is still no recommendation from a scientific society regarding the evaluation of CAC progression. The potential advantage of repeated determinations of CAC may be better for the monitoring of progression over time and may allow to study the impact of exposure to a risk factor. The clinical benefit of CAC progression assessment is demonstrated by the fact that patients with coronary events have a significantly higher rate of CAC progression [23]. A former study suggests that CAC progression rate may also be useful as a predictor of all-cause cardiovascular mortality [24]. Therefore, the potential benefit of CAC progression assessment, similar to 3D volumetric plaque burden testing, could more precisely predict future cardiac events by more accurately estimating the degree of atherosclerosis activity. The development of atherosclerosis does not correspond to a linear function, but can be described as a curve [25]. Single measurements could therefore only insufficiently capture the course of such a curve and thus may not be able to accurately predict the development of atherosclerosis. Multiple measurements of plaque burden and CAC would have the advantage of better mapping the progression of atherosclerosis and enabling more accurate risk stratification.

Another study could demonstrate that patients with PAD had a higher extent of calcification in the coronary arteries and more arterial remodelling [26]. We have to mention that in this study only patients with PAD were examined and no examination of the carotid arteries was made compared to our study.

Patients with peripheral arterial disease (PAD) exhibit a substantially increased risk of subclinical coronary artery disease as well as adverse cardiovascular events compared with individuals without PAD [27]. The coexistence of PAD and coronary artery disease is associated with a markedly worse prognosis, as cardiovascular mortality has been shown to be significantly higher in patients affected by both conditions than in those with only one manifestation of vascular disease. One possible explanation for this observation is the presence of more vulnerable coronary plaques, not only in culprit but also in non-culprit lesions, indicating a more advanced and diffuse atherosclerotic process that may require intensified risk management strategies [28].

In particular, symptomatic PAD represents a strong predictor of cardiovascular morbidity and mortality. Patients with symptomatic disease have been reported to have a markedly elevated risk of cardiovascular events and death compared with individuals without PAD [29]. Furthermore, PAD has been associated with a higher prevalence of complex and severe coronary artery disease, including left main involvement and higher SYNTAX scores [30]. It is also important to note that a considerable proportion of patients with PAD remain clinically asymptomatic, especially those with a reduced ankle–brachial index (<0.9). In this population, a significantly increased prevalence of carotid artery disease has been observed, further supporting the concept of systemic atherosclerosis affecting multiple vascular territories [31].

Another study has demonstrated that coronary vascular dysfunction measured with Positron Emission Tomography Myocardial Perfusion Imaging is frequently observed in patients with peripheral arterial disease and is associated with an increased risk of mortality. These findings further support the concept that atherosclerosis represents a systemic disease process, affecting multiple vascular territories simultaneously. The presence of coronary vascular dysfunction in this patient population may therefore reflect a more advanced and diffuse form of vascular disease, which could contribute to the higher incidence of adverse cardiovascular outcomes [32].

The coronary arteries and the extracranial carotid arteries represent the two vascular territories most frequently involved in cardiovascular events. Previous studies have shown that a proportion of patients present with concomitant coronary and carotid artery disease, with reported prevalence rates ranging between 2% and 14%. Moreover, among patients undergoing coronary artery bypass grafting, approximately 8% have been found to exhibit significant stenosis of the extracranial carotid arteries [33]. Conversely, in patients with high-grade carotid stenosis who are evaluated for surgical intervention, relevant coronary artery stenoses are present in nearly one-third of cases [34]. Although atherosclerosis shares common underlying pathophysiological mechanisms across vascular beds, important differences in plaque characteristics have been described. Features such as plaque erosion, calcified nodules, fibrous cap thickness, and inflammatory cell infiltration may vary between coronary and carotid arteries [35]. A large meta-analysis including more than 20,000 patients investigated the relationship between coronary and carotid atherosclerosis. The results indicated that carotid intima–media thickness increases in parallel with the severity of coronary artery disease [36,37]; at the same time, differences in plaque composition were observed, with a lower prevalence of carotid plaque and calcification but a higher proportion of lipid-rich necrotic core, while intraplaque haemorrhage appeared less frequent [36]. However, no significant differences were found in the overall prevalence of carotid atherosclerosis between patients with and without significant coronary artery disease [37]. Furthermore, carotid intima–media thickness was not consistently associated with the number of affected coronary vessels [38]. In addition, only a moderate relationship has been reported between the extent of stenosis and calcification in carotid and coronary arteries [39]. Notably, carotid and coronary stenosis as well as calcification appear to be stronger predictors of coronary artery disease when intima–media thickness exceeds 1.0 mm, rather than plaque presence alone. Taken together, these findings suggest that while atherosclerosis is a systemic disease affecting multiple vascular territories, its phenotypic expression differs between the coronary and carotid arterial systems.

This study has several limitations that need to be acknowledged. First, the relatively small sample size and the single-centre design may limit the generalizability of the findings. Although we included more patients than originally planned (63 instead of 55) into this study, only 38 completed the follow-up with a second CT scan. Despite the reduced sample size, we were still able to detect statistically significant associations between the progression of coronary artery calcium and peripheral plaque volume. Due to the small sample size, we did not discriminate between stable and unstable plaques in our analysis. Furthermore, as the study population predominantly consisted of individuals with low to intermediate cardiovascular risk, the findings may not be directly transferable to high-risk patient groups.

As the measurement of the CAC in the current clinical setting requires the use of computer tomography, 3D sonography of peripheral atherosclerotic plaques could offer a radiation-free alternative. Furthermore, in contrast to computer tomography, it is significantly cheaper, faster and far more readily available.

Despite the statistical significance of the results, further studies of the analysed correlation with larger numbers of cases are necessary to confirm the findings.

It is unlikely that statin therapy of the included patients (55% of the study population) has influenced our results. The impact of statin therapy on CAC is complex and slower progression under statins as well as increased CAC after long-term use of statins have been reported [40,41,42].

## 5. Conclusions

This prospective observational study demonstrates a significant relationship between coronary and peripheral atherosclerotic burden. Non-invasive imaging of peripheral plaque volume using ultrasound may therefore provide relevant insights into coronary artery disease. Further investigations are required to validate these findings and to explore their clinical implications.

These results underline the potential clinical relevance of combining coronary and peripheral imaging approaches for a more comprehensive assessment of systemic atherosclerosis. In clinical practice, the integration of peripheral plaque assessment may contribute to improved cardiovascular risk stratification, particularly in patients with low to intermediate risk. Furthermore, non-invasive and radiation-free techniques such as 3D ultrasound could serve as a practical tool for longitudinal monitoring of disease progression.

Future studies with larger cohorts are warranted to confirm these findings and to determine whether combined imaging strategies may improve clinical decision-making and patient outcomes.

## Figures and Tables

**Table 1:** Baseline characteristics.

Variable	Study Population *n* = 63	Baseline Group *n* = 25	Follow-Up Group *n* = 38	*p*-Value
Age, years	61.9 [55.34–68.72]	60.2 [55.67–71.22]	63.8 [54.61–68.33]	0.21
Female, *n* (%)	26 (41.27)	8 (32)	18 (47.36)	0.24
Body mass index, kg/m^2^	26.3 [23.00–28.70]	25.1 [23.15–28.55]	26.8 [23.00–28.91]	0.741
Hypertension, *n* (%)	55 (87.3)	13 (52.00)	22 (57.89)	0.645
Family history for CV disease, *n* (%)	19 (30.2)	6 (24)	13 (34.2)	0.444
Smoking, pack years	20 [6.25–38.75]	18.5 [12.50–37.50]	20 [5.00–40.00]	0.365
Hyperlipidaemia, *n* (%)	55 (87.3)	22 (88)	33 (86.8)	0.893
Diabetes mellitus, *n* (%)	3 (4.8)	2 (8.0)	1 (2.6)	0.328
Hs-CRP, mg/dL	0.11 [0.06–0.24]	0.2 [0.06–0.41]	0.11 [0.06–0.16]	0.173
Total cholesterol, mg/dL	179 [151.00–218.00]	197 [159.50–231.50]	176.5 [148.75–215.00]	0.241
LDL-cholesterol, mg/dL	110.5 [85.75–143.75]	114 [88.00–133.00]	109 [84.00–146.00]	0.621
HDL-cholesterol, mg/dL	55 [47.00–69.00]	59 [48.00–76.00]	54 [46.00–66.00]	0.203
Triglyceride, mg/dL	132 [98.00–169.00]	132 [96.50–177.00]	131 [98.50–154.25]	0.922
Creatinine, mg/dL	0.93 [0.84–1.00]	1 [0.84–1.08]	0.92 [0.85–0.99]	0.134
Statin therapy, *n* (%)	35 (55.6)	13 (52.0)	22 (57.9)	0.28
Antihypertensive therapy, *n* (%)	28 (44.4)	10 (40)	18 (47.4)	0.565
Antidiabetic therapy, *n* (%)	2 (3.2)	1 (4.0)	1 (2.6)	0.33
Antiplatelet therapy, *n* (%)	29 (46.9)	11 (44)	18 (47.4)	0.793
Anticoagulation therapy, *n* (%)	5 (7.9)	1 (4)	4 (10.5)	0.348

Data are shown as median [interquartile range] for continuous variables or number (percentage) for categorical variables. CV disease = cardiovascular disease, HDL = high-density lipoprotein, Hs-CRP = high-sensitive C-reactive protein, LDL = low-density lipoprotein.

**Table 2:** Baseline characteristics.

	Study Population *n* = 63	Baseline Group *n* = 25	Follow-Up Group *n* = 38	*p*
Coronary Artery Calcium Score, Agatston Units	73.3 [2.8–324.4]	55.4 [3.7–317.45]	74.7 [0.9–340.95]	0.816
Peripheral Plaque Volume, mm^3^	270 [99–477]	280 [108–492]	269.5 [89.75–479]	0.839

The follow-up examination of the 38 subjects was performed after a median of 24.1 months. The median CAC value of the second examination of the follow-up group was 149.3 Agatston units [IQR 8.48–612.60]. The median peripheral plaque volume was 360.0 mm^3^ [IQR 203.50–604.75].

## Data Availability

The raw data supporting the conclusions of this article will be made available by the authors on request.
